# Comparative Prognostic Performance of CARWL and Naples Prognostic Score in Stage IIIC Non-Small Cell Lung Cancer Treated with Definitive Chemoradiotherapy

**DOI:** 10.3390/medsci14020310

**Published:** 2026-06-12

**Authors:** Erkan Topkan, Duriye Ozturk, Ugur Selek

**Affiliations:** 1 Department of Radiation Oncology, Faculty of Medical, Baskent University, Adana 01120, Turkey; 2 Department of Radiation Oncology, Faculty of Medicine, Afyonkarahisar Health Sciences University, Afyonkarahisar 03030, Turkey; duriyeozturk07@gmail.com; 3 Department of Radiation Oncology, School of Medicine, Koc University, Istanbul 34010, Turkey; ugurselek@yahoo.com

**Keywords:** carcinoma, non-small-cell lung, chemoradiotherapy, prognosis, CARWL score, Naples prognostic score

## Abstract

Background: Prognostic stratification remains challenging in patients with stage IIIC non-small cell lung cancer (NSCLC) treated with definitive chemoradiotherapy (CCRT), and the relative performance of host-related prognostic indices in this setting is unclear. The CARWL (C-reactive Protein, Albumin, and Recent Weight Loss) score and the Naples prognostic score (NPS) have each been proposed as prognostic tools, but direct comparisons are lacking. This study compared their prognostic performance. Methods: We retrospectively analyzed 795 patients with stage IIIC NSCLC treated with CCRT between 2010 and 2020. Patients were stratified into three prognostic groups according to CARWL and NPS. Overall survival (OS) was the primary endpoint; progression-free survival (PFS) and locoregional PFS (LRPFS) were secondary endpoints. Survival was assessed using Kaplan–Meier analysis and Cox regression. Results: Both CARWL and NPS significantly stratified OS, PFS, and LRPFS (all *p* < 0.001). CARWL demonstrated modestly higher discriminatory performance across endpoints. The OS difference between unfavorable and favorable groups was larger with CARWL than with NPS (19.3 vs. 12.3 months). CARWL also provided greater separation for PFS (5.3 vs. 3.2 months) and LRPFS (4.9 vs. 3.4 months). In multivariable analyses, both indices retained independent prognostic significance; however, CARWL consistently exhibited stronger hazard gradients and maintained prognostic value when modeled alongside NPS. Conclusions: Both CARWL and NPS offered meaningful prognostic stratification in stage IIIC NSCLC treated with CCRT, but CARWL demonstrated a modest but more consistent prognostic discrimination than NPS. Pending external validation, CARWL represents a practical and biologically grounded tool for risk stratification in this population.

## 1. Introduction

Despite recent advances in diagnostic, therapeutic, and supportive care practices, the prognosis for stage IIIC non-small cell lung cancer (NSCLC) patients treated with standard definitive concurrent chemoradiotherapy (CCRT) remains dismal, with 5-year expected overall survival (OS) rates between only 10 and 15% [1,2]. Adjuvant immunotherapy administered after definitive CCRT has improved survival durations in a subset of patients who responded to CCRT [3,4]. However, health insurance restrictions due to the high cost of adjuvant immunotherapy pose a problem in many countries, discouraging its routine use.

The emergence of distant metastases and/or uncontrollable local/regional recurrences is the primary cause of unfavorable treatment outcomes following radical CCRT regimens, largely attributable to resistance to radiotherapy and existing chemotherapeutic agents [5,6]. Interestingly, even identical CCRT regimens frequently yield significantly different rates of locoregional or distant tumor control and varying survival durations among patients, despite similar distributions of initial patient and disease characteristics. These pronounced discrepancies in outcomes among patients at the same stage indicate that the current TNM (tumor-node-metastasis) staging system falls short in accurately stratifying prognosis. This inadequacy arises because the TNM staging framework focuses primarily on tumor size and its local and regional extension, while neglecting crucial elements such as the tumor’s microenvironment, genetics, and other biological factors [6,7]. For instance, the existing TNM staging system classifies all tumors larger than 7 cm as T4, despite significant variability in volumetric change and associated tumor cell load across different tumor sizes, leading to disparate tumor control rates even with the same CCRT regimens. Therefore, integrating new biological markers with tumor stage data could plausibly enhance prognostic stratification and enable more tailored management strategies for these patients.

Chronic systemic inflammation, recognized as the seventh hallmark of carcinogenesis, not only facilitates carcinogenic cellular transformation but also promotes tumor cell proliferation, growth, invasion, neoangiogenesis, survival, immune evasion, metastasis, and resistance to apoptosis [8]. Within this framework, the C-reactive protein-to-albumin ratio (CAR) has emerged as a robust prognostic biomarker, demonstrating value in stratifying patients across various disease stages and treatment modalities [9,10,11]. Significant weight loss (WL), which is present to some degree in up to 80% of all NSCLC patients and represents another adverse prognostic factor strongly linked to cancer cachexia in locally advanced NSCLC (LA-NSCLC), has likewise been associated with persistent inflammation and related biomarkers [12,13,14]. In our recent research, we proposed the novel CARWL score, which combines CAR and significant WL, and demonstrated its ability to classify LA-NSCLC patients into three distinct prognostic categories—low, intermediate, and high risk—based on survival outcomes [15]. Moreover, the Naples prognostic score (NPS), which incorporates pretreatment albumin, total cholesterol, neutrophil-to-lymphocyte ratio (NLR), and monocyte-to-lymphocyte ratio (MLR), has also been shown to effectively stratify patients with LA-NSCLC into three survival groups [16,17]. While both indices serve as useful prognostic tools, the CARWL score and the NPS differ fundamentally, sharing only albumin.

Given the previously reported prognostic relevance of both indices in LA-NSCLC, a direct comparative evaluation may help clarify their relative discriminatory performance. To address this gap in the literature, we conducted a retrospective analysis of 795 patients with stage IIIC NSCLC treated with definitive CCRT, comparing the prognostic performance of the CARWL score and NPS.

## 2. Patients and Methods

### 2.1. Study Population

We retrospectively analyzed patients recorded in a prospectively maintained institutional database. During routine clinical care, demographic, clinical, laboratory, treatment, and follow-up data were recorded according to institutional standards. Eligibility criteria, including age 18–80 years and BMI > 18.5 kg/m^2^, reflected institutional criteria for curative-intent definitive CCRT and were applied to maintain a clinically homogeneous cohort and reduce confounding related to frailty, severe undernutrition, and treatment tolerance. Eligible patients met the following criteria: age 18–80 years, Eastern Cooperative Oncology Group (ECOG) performance score 0–1, body mass index (BMI) > 18.5 kg/m^2^; histologically confirmed NSCLC (adenocarcinoma or squamous cell carcinoma); stage IIIC disease determined by computed tomography (CT) and 18F-fluorodeoxyglucose positron emission tomography-CT (PET-CT); available pre-CCRT brain magnetic resonance imaging; and complete blood count and biochemical test results. Exclusion criteria included malignant pleural or pericardial effusion, involvement of contralateral supraclavicular lymph nodes, prior radiotherapy or chemotherapy, and insufficient pulmonary, cardiac, renal, or hepatic function. Additionally, patients with active infectious diseases or those receiving immunosuppressive therapy within 30 days prior to the initiation of CCRT were excluded to minimize potential biases affecting immune and inflammatory markers, and consequently, patient outcomes.

### 2.2. Treatment Protocol

Thoracic RT plans were generated for each patient using co-registered diagnostic CT and PET-CT images, in accordance with our institutional care standards. RT was delivered with megavoltage linear accelerators using the intensity-modulated RT (IMRT) technique. Definitions of target volumes, total and fractional doses, normal tissue dose constraints, and concurrent chemotherapy regimens were consistent with our previous report [18]. All patients received 60 Gy of IMRT in 30 fractions (2 Gy per fraction, 5 days per week) and concurrently received 1–3 cycles of cisplatin or carboplatin, combined with docetaxel, paclitaxel, or vinorelbine, during the RT course (every 21 days). In line with institutional policy, elective thoracic nodal irradiation was not performed in any patient. Chemotherapy selection was protocol-driven and followed institutional multidisciplinary treatment guidelines. Cisplatin-based regimens were preferred whenever clinically appropriate, whereas carboplatin-containing regimens were used when cisplatin was contraindicated or deemed unsuitable due to patient-specific clinical factors. Regimen selection additionally considered performance status, comorbidities, organ function, and anticipated treatment tolerance.

### 2.3. Measures of CARWL Score and Naples Prognostic Score

The novel CARWL score is a three-tiered immune–inflammation–nutrition index, recently developed by Topkan et al., that divides patients into three risk groups (Table 1) [15]. The CARWL score was developed by combining the pretreatment CAR, which was calculated using CRP and albumin measures obtained on the first day of CCRT: CAR = CRP/Albumin, and significant WL, defined as WL > 5% in the previous six months according to the Delphi consensus [19]. Pretreatment WL was ascertained from prospectively maintained institutional records by comparing body weight at CCRT initiation with the documented body weight within the preceding six months, and values were verified during routine pretreatment clinical and nutritional assessment [15]. The NPS, first proposed by Galizia et al. as a novel prognostic tool for patients with colorectal cancer [20], is based on four parameters: serum albumin, total cholesterol, NLR, and LMR (Table 1). The NPS stratifies patients into three risk categories, similar to the more recently developed CARWL score. As with the CARWL score, all parameters required for NPS calculation were obtained on the first day of CCRT.

### 2.4. Follow-Up and Treatment Response Evaluations

Although the study was retrospective in design, patient evaluations were recorded prospectively. After completing CCRT, patients attended follow-up visits on a predetermined schedule: every three months during the first two years, and every six months thereafter or more frequently if clinically indicated. Therapeutic response was assessed using serial complete blood counts, biochemistry tests, and PET-CT or chest CT scans, in accordance with the European Organization for Research and Treatment of Cancer (EORTC) 1999 guidelines, with chest CT replacing PET-CT after confirmation of a complete metabolic response. Additional imaging from radiology or nuclear medicine was reserved for cases with clinical suspicion of locoregional recurrence or distant metastasis.

### 2.5. Statistical Analysis

A complete-case analysis approach was used; therefore, patients with missing data required for eligibility assessment, CARWL or NPS calculation, treatment characterization, or survival analyses were excluded, and no imputation procedures were performed. The primary objective of this retrospective study was to compare the prognostic performance of the CARWL score and the NPS for OS, defined as the interval from the initiation of CCRT to death from any cause or last follow-up. Secondary endpoints included locoregional progression-free survival (LRPFS), defined as the time from treatment initiation to locoregional recurrence or progression involving the primary tumor site and/or ipsilateral or contralateral hilar or mediastinal lymph nodes, and progression-free survival (PFS), defined as the interval from the first day of CCRT to the first documented disease progression, death, or last follow-up. Continuous variables were summarized using medians and ranges, while categorical variables were reported as frequencies and percentages. Comparisons between groups were performed using the chi-square test or Fisher’s exact test for categorical variables, and Student’s *t*-test or the Mann–Whitney U test for continuous variables, as appropriate based on data distribution. For comparisons involving three or more groups, parametric or nonparametric methods were applied accordingly, with adjustment for multiple testing.

For comparative analyses, the CARWL score and NPS were each stratified into three predefined risk groups according to previously published definitions. Survival curves were estimated using the Kaplan–Meier method and compared using log-rank tests. Univariate analyses were initially performed to identify variables associated with survival outcomes, and variables demonstrating statistical significance were subsequently entered into multivariate Cox proportional hazards regression models to assess independent prognostic significance. The primary multivariable Cox models included variables that were statistically significant in univariate analyses. In addition, sensitivity multivariable models including clinically relevant available covariates were performed to assess the robustness of the associations between CARWL, NPS, and survival outcomes. The proportional hazards assumption was evaluated and found not to be violated. To quantitatively assess and compare the discriminatory ability of CARWL and NPS for OS, Harrell’s concordance index (C-index) was calculated from index-only Cox regression models, with each score included separately. Incremental discrimination was quantified by calculating the difference in C-indices (ΔC-index). In addition, adjusted Cox models incorporating established clinical covariates were constructed to evaluate the incremental prognostic value of each index in a clinical context, and corresponding adjusted C-indices were reported. Likelihood-based model comparisons were restricted to nested models, and no formal statistical testing was applied to non-nested C-index differences. Internal validation was performed using bootstrap resampling to estimate confidence intervals for discrimination metrics. All statistical tests were two-sided, with *p* < 0.05 considered statistically significant for two-group comparisons; for three-group comparisons, Bonferroni-corrected *p* values < 0.0167 were considered statistically significant. All analyses were conducted with prespecified endpoints and without post hoc modification of score definitions or cut-off values, to minimize model optimism and preserve the interpretability of the comparative results.

## 3. Results

### 3.1. Patient Characteristics

A total of 795 consecutive patients with stage IIIC NSCLC treated with definitive CCRT between 2010 and 2020 were included in the analysis. The median age was 65 years (range, 27–79), with 70.6% of patients aged ≤70 years and 29.4% older than 70 years. Most patients were male (63.8%), ever smokers (94.8%), and had an Eastern Cooperative Oncology Group (ECOG) performance status of 1 (73.6%). Adenocarcinoma was the predominant histological subtype (61.0%), and T3 disease was more frequent than T4 disease (55.7% vs. 44.3%). Concurrent chemotherapy consisted of cisplatin- or carboplatin-based combinations with docetaxel, paclitaxel, or vinorelbine. Most patients received three cycles of concurrent chemotherapy. The distributions of chemotherapy regimens and chemotherapy cycles were well balanced across both CARWL and NPS groups (Table 1).

Treatment was generally well tolerated. Acute grade 3 and grade 4 toxicities occurred in 259 (32.6%) and 43 (5.4%) patients, respectively, while no grade 5 acute toxicities were observed. Regarding late adverse events, grade 3–4 toxicities were documented in 68 patients (8.6%), and fatal (grade 5) toxicities occurred in 8 patients (1.0%). Causes of late treatment-related deaths included intractable broncho-esophageal fistula (*n* = 3), massive pulmonary hemoptysis (*n* = 3), and radiation-induced pneumonia (*n* = 2).

### 3.2. Survival Outcomes According to CARWL Score

**Figure 1:**
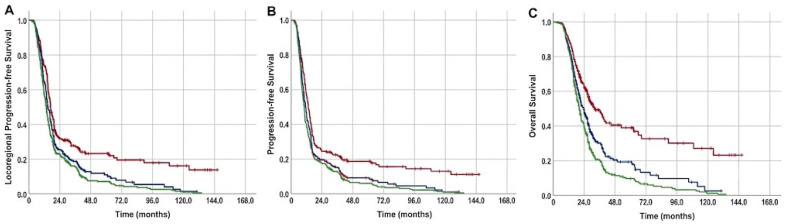
Survival outcomes according to CARWL score groups. (**A**) Locoregional progression-free survival (LRPFS), (**B**) progression-free survival (PFS), and (**C**) overall survival (OS). CARWL-0 represents the most favorable prognostic group, CARWL-1 the intermediate-risk group, and CARWL-2 the least favorable prognostic group. Survival distributions were compared using the log-rank test. Color codes: red, CARWL-0; dark blue, CARWL-1; green, CARWL-2.

The CARWL score demonstrated clear and consistent stratification of survival outcomes across its three predefined categories (Figure 1). Median LRPFS was 17.8, 14.9, and 12.9 months for the CARWL-0, CARWL-1, and CARWL-2 groups, respectively (log-rank *p* < 0.001). Median PFS followed a similar pattern, with values of 14.6, 11.2, and 9.3 months (log-rank *p* < 0.001). OS showed the most pronounced separation, with median OS durations of 36.6 months for CARWL-0, 23.9 months for CARWL-1, and 17.3 months for CARWL-2 (log-rank *p* < 0.001).

### 3.3. Survival Outcomes According to Naples Prognostic Score

**Figure 2:**
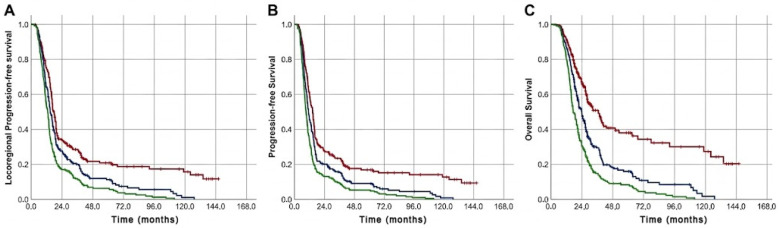
Survival outcomes according to Naples Prognostic Score (NPS) groups. (**A**) Locoregional progression-free survival (LRPFS), (**B**) progression-free survival (PFS), and (**C**) overall survival (OS). NPS-0 represents the most favorable prognostic group, NPS-1 the intermediate-risk group, and NPS-2 the least favorable prognostic group. Survival distributions were compared using the log-rank test. Color codes: red, NPS-0; dark blue, NPS-1; green, NPS-2.

The NPS also demonstrated effective stratification of survival outcomes, although the degree of separation was less pronounced than that observed with CARWL (Figure 2). Median LRPFS durations were 16.7, 14.7, and 13.3 months for the NPS-0, NPS-1, and NPS-2 groups, respectively (log-rank *p* = 0.002). Median PFS values were 13.4, 11.4, and 10.2 months (*p* = 0.003), while median OS durations were 32.5, 23.6, and 20.2 months, respectively (*p* < 0.001).

### 3.4. Comparative Performance of CARWL and NPS

Baseline clinicopathological characteristics were well balanced across CARWL and NPS subgroups with respect to age, sex, ECOG performance status, smoking history, histology, and T stage (Table 2), with no statistically significant differences observed (all *p* > 0.05). Both CARWL and NPS effectively stratified patients into three prognostic risk groups across all survival endpoints. However, direct comparison demonstrated that CARWL provided modestly higher discriminatory performance, particularly for OS.

The difference in median OS between the most unfavorable and most favorable groups was 19.3 months for CARWL (17.3 vs. 36.6 months; *p* < 0.001; hazard ratio [HR]: 2.47; 95% confidence interval [CI]: 1.93–3.28), compared with 12.3 months for NPS (20.2 vs. 32.5 months; *p* < 0.001; HR: 1.72; 95% CI: 1.41–2.14). CARWL also yielded greater separation for PFS, with a 5.3-month difference between CARWL-2 and CARWL-0 (9.3 vs. 14.6 months; HR: 1.98; 95% CI: 1.63–2.42), whereas the corresponding difference for NPS was 3.2 months (10.2 vs. 13.4 months; HR: 1.55; 95% CI: 1.27–1.88). Similarly, LRPFS separation was more pronounced with CARWL (12.9 vs. 17.8 months; difference 4.9 months; HR: 1.81; 95% CI: 1.39–2.38) than with NPS (13.3 vs. 16.7 months; difference 3.4 months; HR: 1.52; 95% CI: 1.25–1.86).

### 3.5. Discrimination Analyses

**Figure 3:**
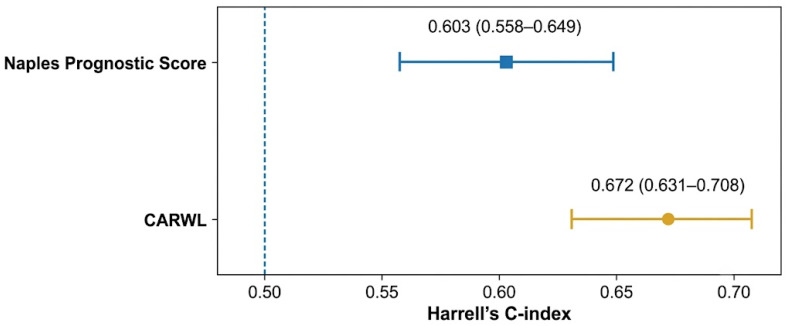
Discriminatory performance of CARWL and Naples Prognostic Score (NPS) for overall survival. Harrell’s concordance indices (C-index) with 95% confidence intervals were derived from index-only Cox proportional hazards models. Higher C-index values indicate greater prognostic separation. The dashed vertical line at C = 0.50 denotes the level expected by chance alone.

In discrimination analyses for OS, CARWL demonstrated modestly higher prognostic concordance than NPS. Using index-only Cox proportional hazards models, Harrell’s concordance index was numerically higher for CARWL than for NPS. Specifically, the C-index for CARWL was 0.672 (95% CI, 0.631–0.708), whereas the corresponding C-index for the NPS was 0.603 (95% CI, 0.558–0.649), yielding a ΔC-index of 0.069 in favor of CARWL (Figure 3). In adjusted Cox models that incorporated T stage, the established clinical covariate identified in univariate analyses, CARWL consistently showed higher concordance than NPS, supporting its incremental prognostic value beyond tumor burden alone. Internal validation using bootstrap resampling (1000 iterations) confirmed the stability of these discrimination estimates. No formal statistical testing was applied to the observed ΔC-index, as comparisons of C-indices between non-nested models are inherently descriptive.

### 3.6. Univariate and Multivariate Analysis

In univariate analyses (Table 3), T stage, CARWL score, and NPS were significantly associated with LRPFS, PFS, and OS (all *p* < 0.05 for T stage; all Bonferroni-corrected *p* < 0.0167 for CARWL score and NPS). Multivariate Cox regression models incorporating T stage, CARWL score, and NPS confirmed that all three variables were independently associated with LRPFS, PFS, and OS (all *p* < 0.05 for T stage; all Bonferroni-corrected *p* < 0.0167 for CARWL score and NPS). In sensitivity multivariable analysis for OS, additionally incorporating age, sex, ECOG performance status, smoking history, histology, chemotherapy cycles, and radiotherapy interruption, both CARWL and NPS remained independently associated with OS (Supplementary Table S2).

## 4. Discussion

This large retrospective cohort of 795 patients with stage IIIC non-small cell lung cancer treated with definitive chemoradiotherapy was used to compare the relative prognostic robustness of the novel CARWL score and the Naples prognostic score (NPS). Both indices independently stratified patients into three distinct outcome groups and retained prognostic significance across survival endpoints. Notably, the CARWL score demonstrated a modest but consistent improvement in discriminatory capacity for overall survival, locoregional progression-free survival, and progression-free survival compared with the NPS. This finding underscores the prognostic relevance of systemic immunity, persistent inflammation, and cancer-associated weight loss, which are integrally captured within the CARWL construct. Importantly, such incremental gains in discrimination are typical when comparing clinically applicable host-related prognostic indices in advanced oncologic cohorts.

In addition to reaffirming the well-established prognostic significance of T-stage in patients with LA-NSCLC [21], our study provides compelling evidence for the independent prognostic value of both the CARWL score and the NPS in effectively stratifying NSCLC patients into three distinct prognostic groups [15,20]. However, the most notable finding of our study was the greater separation between risk categories achieved with the CARWL score than with the NPS. The difference in median OS between the best and worst strata was 19.3 months with the CARWL score, compared with 12.3 months with the NPS. Similarly, the CARWL score showed greater risk separation for PFS (5.3 vs. 3.2 months) and LRPFS (4.9 vs. 3.4 months). Hazard ratios were also consistently higher for CARWL score groups (Table 3), reflecting a stronger predictive gradient. Of note, the CARWL score retained its independent prognostic significance for the outcomes, whereas the prognostic influence of NPS, although statistically significant, was somewhat reduced in the multivariate analysis including both indices, suggesting that the CARWL score may be more potent in capturing biologically relevant determinants of disease prognosis than NPS. Moreover, these results were observed with well-balanced distributions of baseline patient and disease characteristics between the CARWL and NPS subgroups (Table 1), indicating that the differences in survival outcomes were primarily attributable to the prognostic indices rather than to baseline imbalances. These findings support the concept that host-related inflammatory, nutritional, and weight-loss-related factors may provide prognostic information beyond conventional anatomic staging alone.

Previous studies have independently validated the CAR [9,10], significant WL [12,13], and NPS [16,17] as adverse prognostic factors in LA-NSCLC. The components of the CARWL score—CAR and significant WL—have consistently been linked to poorer survival outcomes across various treatment settings in LA-NSCLC [16,17,22,23]. Furthermore, our recent research, which introduced the CARWL score to the literature, demonstrated its robustness as a tool for stratifying LA-NSCLC patients into three distinct prognostic categories: low, intermediate, and high risk—based on survival outcomes [15]. Similarly, the NPS, initially developed for colorectal cancer [20], has shown prognostic relevance in LA-NSCLC patients by classifying them into three survival groups [24,25]. Our findings extend these observations by providing the first direct comparison between the CARWL score and NPS in a homogeneously treated cohort of patients with stage IIIC NSCLC, suggesting that CARWL may offer modestly improved prognostic stratification compared with NPS in this population. Because CARWL and NPS are established prognostic scoring systems with predefined three-tier classifications, the present study retained their original group structures to preserve methodological consistency with prior studies and enable direct comparative evaluation. Nevertheless, risk separation was most pronounced between the favorable category and the remaining categories, suggesting that the strongest clinical discrimination of both indices may occur at the lower-risk boundary.

Consistent with these findings, CARWL also exhibited modestly higher discriminatory performance for OS as quantified by Harrell’s C-index. Beyond the clear separation observed in Kaplan–Meier analyses and the steeper hazard gradients identified in Cox regression models, CARWL demonstrated higher concordance than the NPS. Although the absolute improvement in concordance was modest, the observed ΔC-index is consistent with differences typically seen between competing clinical prognostic indices in advanced oncology cohorts, where gains in discrimination are often incremental rather than transformative. Importantly, CARWL retained its prognostic advantage in models adjusted for tumor burden, supporting its incremental value beyond established disease-related factors and underscoring the relevance of host-related inflammatory and nutritional status in shaping outcomes following chemoradiotherapy. In this context, even moderate improvements in discrimination may be clinically meaningful in advanced thoracic malignancies, where substantial gains are rarely achieved with inexpensive, routinely available biomarkers alone.

The mechanisms underlying the improved prognostic discrimination of CARWL relative to NPS remain uncertain, as similarly designed comparative and mechanistic studies are currently unavailable. Nevertheless, this difference may be related to CARWL’s ability to integrate two complementary domains: systemic inflammation/nutritional reserve, as reflected by CAR, and clinically evident weight-loss dynamics. Elevated CAR is associated with increased CRP and reduced albumin, a combination that reflects inflammation, impaired nutritional status, immunosuppression, tumor aggressiveness, impaired treatment tolerance, and resistance to chemo- and radiotherapy [9,26]. Significant WL, meanwhile, is a core clinical feature of cancer cachexia and has been associated with reduced physical performance, poorer treatment compliance, and inferior oncological outcomes across cancer types, including locally advanced NSCLC treated with definitive CCRT [19,27,28,29,30]. Supporting this biological plausibility, both CAR and the related Glasgow Prognostic Score have been associated with worse survival in NSCLC [9,11], while CRP and albumin have also been identified as biochemical markers relevant to definitions and classification frameworks of cachexia [31,32]. Therefore, by combining CAR with significant WL, CARWL may capture a broader host-related vulnerability phenotype than NPS, which incorporates albumin, cholesterol, NLR, and LMR but does not directly include weight-loss dynamics. Importantly, the prognostic value of CARWL did not appear to be explained solely by measurable differences in treatment delivery, as chemotherapy cycles delivered, radiotherapy interruption rates and duration, hospitalization during CCRT, and acute grade 3–4 toxicities were comparable across CARWL groups (Supplementary Table S1). However, this interpretation remains hypothesis-generating, and future clinical and mechanistic studies are required to determine whether CARWL reflects cachexia-related biology, treatment tolerance, tumor aggressiveness, or a combination of these processes.

From a clinical standpoint, patients with identical disease stages and comparable treatments may still experience markedly different outcomes, supporting the need for practical prognostic stratification in LA-NSCLC. Because CARWL relies on simple, inexpensive, and routinely available clinical and laboratory parameters, it may help identify patients at higher risk of treatment failure without adding financial or logistical burden. At present, CARWL should be viewed primarily as a prognostic stratification tool rather than a treatment-selection biomarker. If validated in future studies, CARWL may be used alongside TNM staging to support closer surveillance, earlier integration of supportive care, and stratification in clinical trials evaluating personalized treatment strategies.

This study is strengthened by its large cohort size, consistent staging procedure incorporating FDG-PET-CT in all patients, uniform CCRT protocol, specific focus on stage IIIC patients, and extended follow-up duration, which collectively minimize heterogeneity and enhance the reliability of the comparisons made. However, several limitations should be acknowledged. First, despite the prospective acquisition of clinical and laboratory data, the retrospective nature of the study introduces the possibility of residual confounding and inherent selection bias. Second, because CARWL was originally developed by our research group, the present study should be regarded as an additional institutional validation rather than an independent external validation. Therefore, confirmation in independent, multi-institutional cohorts is necessary to establish the reproducibility and generalizability of our findings, particularly in contemporary cohorts treated with consolidation durvalumab following definitive CCRT. Because the present study focused primarily on comparative discrimination rather than individualized risk prediction, formal calibration analyses were not performed. Third, as only patients with an ECOG performance status of 0–1 who received a relatively uniform treatment protocol were included, the results may not fully reflect the broader population of stage IIIC NSCLC patients encountered in routine practice. Furthermore, detailed volumetric and metabolic tumor-burden parameters, including gross tumor volume, planning tumor volume, metabolic tumor volume, and total lesion glycolysis, were not consistently available and therefore could not be evaluated. Consequently, we were unable to determine whether the prognostic value of CARWL is independent of these more refined measures of tumor burden. Additionally, detailed pulmonary function parameters and comorbidity burden were not consistently available for inclusion in the multivariable models, and residual confounding from unmeasured clinical factors cannot be ruled out. Fourth, although substantial temporal variations in the individual CARWL and NPS components may occur during CCRT and follow-up owing to their dynamic biological nature, all analyses were based on single pretreatment measurements. Fifth, potential variations in salvage therapies during follow-up may have influenced survival outcomes and thereby affected the relative prognostic performance of CARWL and NPS. Sixth, although CARWL incorporates systemic inflammation and clinically significant weight loss, it does not account for other potentially relevant prognostic determinants such as genomic alterations, immune checkpoint expression, or tumor microenvironmental characteristics, largely reflecting the limited availability of specialized molecular testing during much of the study period. Whether the prognostic performance of CARWL persists in immunotherapy-integrated treatment paradigms, particularly among patients receiving consolidation durvalumab after definitive CCRT, warrants further investigation. Given the interactions among systemic inflammation, nutritional status, host immunity, and immunotherapy outcomes, future validation studies should specifically evaluate CARWL in contemporary PACIFIC-era cohorts. Therefore, the present findings should be regarded as hypothesis-generating rather than definitive until confirmed by prospective investigations specifically designed to address these limitations.

## 5. Conclusions

In conclusion, this retrospective cohort analysis indicates that both the CARWL score and NPS are independent predictors of survival outcomes in stage IIIC NSCLC patients treated with definitive CCRT. However, the CARWL score showed modestly greater prognostic discrimination than NPS for LRPFS, PFS, and OS. By jointly capturing systemic inflammation, nutritional status, and clinically significant weight loss, CARWL represents an inexpensive and clinically practical tool for prognostic stratification. If validated in independent cohorts, its integration with conventional staging may help refine risk assessment and support risk-adapted management strategies in this patient population.

## Figures and Tables

**Table 1:** Pretreatment patient and disease characteristics at presentation.

Covariate	All Patients (*n* = 795)	CARWL-0 (*n* = 249)	CARWL-1 (*n* = 287)	CARWL-2 (*n* = 259)	*p*-Value	NPS-0 (*n* = 219)	NPS-1 (*n* = 239)	NPS-2 (*n* = 337)	*p*-Value
Median age, y (range)	65 (27–79)	65 (29–79)	64 (32–79)	66 (27–78)	0.86	64 (31–79)	65 (36–78)	66 (27–79)	0.91
Age group, y (%)									
≤70 years	561 (70.6)	185 (74.3)	190 (66.2)	186 (71.8)	0.32	156 (71.2)	168 (70.3)	237 (70.4)	0.67
>70 years	234 (29.4)	64 (25.7)	97 (33.8)	73 (28.2)		63 (28.8)	71 (29.7)	100 (29.6)	
Gender, *n* (%)									
Female	288 (36.2)	91 (36.5)	102 (35.5)	95 (36.7)	0.83	80 (36.5)	88 (36.8)	120 (35.6)	0.77
Male	507 (63.8)	287 (63.5)	227 (64.5)	164 (63.3)		139 (63.5)	151 (63.2)	217 (64.4)	
ECOG, *n* (%)									
0	210 (26.4)	65 (26.1)	74 (25.8)	71 (27.4)	0.74	58 (26.5)	65 (27.2)	87 (25.8)	0.53
1	585 (73.6)	184 (73.9)	213 (74.2)	188 (72.6)		161 (73.5)	174 (72.8)	250 (74.2)	
Smoking history, *n* (%)									
Absent	41 (5.2)	12 (4.8)	15 (5.2)	14 (5.4)	0.87	12 (5.5)	13 (5.4)	16 (4.7)	0.82
Present	754 (94.8)	237 (95.2)	272 (94.8)	245 (94.6)		207 (94.5)	226 (94.6)	321 (95.0)	
Histology, *n* (%)									
SCC	310 (39.0)	101 (40.6)	106 (36.9)	103 (39.8)	0.49	83 (37.9)	94 (39.3)	133 (39.5)	0.73
AC	485 (61.0)	148 (59.4)	181 (63.1)	156 (60.2)		136 (62.1)	145 (60.7)	204 (60.5)	
T-stage, *n* (%)									
3	443 (55.7)	140 (56.2)	155 (54.0)	148 (57.3)	0.56	124 (56.6)	138 (57.7)	181 (53.7)	0.58
4	352 (44.3)	109 (43.8)	132 (46.0)	111 (42.7)		95 (43.4)	101 (42.3)	156 (46.3)	
Chemotherapy regimen, *n* (%)									
Cisp. + docetaxrel	264	84	92	88	0.72	78	81	105	0.47
Cisp. + paclitaxel	122	36	47	39		27	31	64	
Cisp. + vinorelbin	108	32	40	36		31	30	47	
Carbo. + docetaxrel	159	48	59	52		46	49	64	
Carbo. + paclitaxel	75	21	29	25		22	23	30	
Carbo. + vinorelbin	67	28	20	29		15	25	27	
Chemotherapy cycles, *n* (%)									
1	84	26	31	27	0.59	23	25	36	0.64
2	129	42	47	40		41	42	46	
3	582	181	209	192		155	172	255	

Abbreviations: CARWL: Combination of C-reactive-protein-to-albumin ratio (CAR) and significant weight loss (WL); NPS: Naples prognostic score; ECOG: Eastern Cooperative Oncology Group; SCC: Squamous-cell carcinoma; AC: Adenocarcinoma; T-stage: Tumor stage; Cisp.: Cisplatin; Carbo.: Carboplatin. Note: Age was categorized using the clinically established threshold of 70 years, which is commonly used in thoracic and geriatric oncology research.

**Table 2:** Definitions of CARWL score and Naples Prognostic score groups.

Group	Definition
CARWL score	
CARWL-0	CAR < 3.0 and WL ≤ 5.0%
CARWL-1	CAR < 3.0 and WL > 5.0% or CAR ≥ 3.0 and WL ≤ 5.0%
CARWL-2	CAR > 3.0 and WL > 5.0%
NPS *	
NPS-0	0 point
NPS-1	1 or 2 points
NPS-2	3 or 4 points

Abbreviations: CARWL: Combination of C-reactive-protein-to-albumin ratio (CAR) and significant weight loss (WL); NPS: Naples prognostic score. * Naples prognostic score groups patients according to four factors: albumin, ≥40 g/L (0 point) or <40 g/L (1 point); total cholesterol, >180 mg/dL (0 point) or ≤180 mg/dL (1 point); neutrophil-to-lymphocyte ratio (NLR), ≤2.96 (0 point) or >2.96 (1 point); and lymphocyte-to-monocyte ratio (LMR), >4.44 (0 point) or ≤4.44 (1 point).

**Table 3:** Univariate and multivariate analyses of outcomes.

Characteristic	Patients (*n* = 795)	Median LRPFS (Months)	Univariate *p*-Value	Multivariate *p*-Value	Median PFS (Months)	Univariate *p*-Value	Multivariate *p*-Value	Median OS (Months)	Univariate *p*-Value	Multivariate *p*-Value
Age group										
≤70 years	561	15.6	0.44	-	11.6	0.39	-	24.3	0.29	-
>70 years	234	14.1			10.7			22.7		
Gender										
Female	288	14.2	0.49	-	10.8	0.53	-	22.6	0.48	-
Male	507	15.6			11.5			24.1		
ECOG										
0	210	15.5	0.71	-	11.4	0.62	-	23.9	0.82	-
1	585	14.6			10.6			23.1		
Smoking history, *n* (%)										
Absent	41	16.0	0.58	-	11.7	0.72	-	25.4	0.38	-
Present	754	14.6			11.1			23.3		
Histology										
SCC	310	13.8	0.27	-	10.6	0.41	-	22.8	0.54	-
AC	485	15.4			11.7			24.2		
T-stage										
3	443	16.5	0.007	0.009	13.2	0.004	0.002	26.8	0.006	0.001
4	352	13.3			10.7			21.3		
CARWL score group										
0	249	17.8	<0.001 *	<0.001 *	14.6	<0.001 *	<0.001 *	36.6	<0.001 *	<0.001 *
1	287	14.9			11.2			23.9		
2	259	12.9			9.3			17.3		
NPS group										
0	219	16.7	0.002 *	0.004 *	13.4	0.003 *	0.005 *	32.5	<0.001 *	<0.001 *
1	239	14.7			11.4			23.6		
2	337	13.3			10.2			20.2		

Abbreviations: LRPFS: Locoregional progression-free survival; PFS: Progression-free survival; OS: Overall survival; ECOG: Eastern Cooperative Oncology Group; SCC: Squamous-cell carcinoma; AC: Adenocarcinoma; T-stage: Tumor stage; CARWL: Combination of C-reactive-protein-to-albumin ratio (CAR) and significant weight loss (WL); NPS: Naples prognostic score. * A significant Bonferroni-corrected *p*-value for comparisons between three groups should be <0.0167.

## Data Availability

The original contributions presented in this study are included in the article/Supplementary Material. Further inquiries can be directed to the corresponding author.

## Supplementary Materials

The following supporting information can be downloaded at: https://www.mdpi.com/article/10.3390/medsci14020310/s1, Table S1: Treatment delivery and completion according to CARWL score groups, Table S2: Sensitivity multivariable Cox regression analysis for overall survival.
