# Supplementary material for: Comparative Prognostic Performance of CARWL and Naples Prognostic Score in Stage IIIC Non-Small Cell Lung Cancer Treated with Definitive Chemoradiotherapy
Source: Med Sci (Basel). 2026 Jun 12;14(2):310. doi: 10.3390/medsci14020310 (PMC13304127; doi:10.3390/medsci14020310)
Supplement: Supplementary file 1 [file medsci-14-00310-s001.zip › medsci-4365086-supplementary.pdf]

Supplementary Tables

Table S1. Treatment delivery and completion according to CARWL score groups.

Treatment variable	All patients (N=795)	CARWL-0 (N=249)	CARWL-1 (N=287)	CARWL-2 (N=259)	P-value
Chemotherapy cycles delivered, n (%)					
1 cycle	84 (10.6)	26 (10.4)	31 (10.8)	27 (10.4)	0.92
2 cycles	129 (16.2)	42 (16.9)	47 (16.4)	40 (15.4)	
3 cycles	582 (73.2)	181 (72.7)	209 (72.8)	192 (74.1)	
RT interruption, n (%)					
No	706 (88.8)	222 (89.6)	257 (89.2)	227 (87.6)	0.59
Yes	89 (11.2)	26 (10.4)	31 (10.8)	32 (12.4)	
*RT interruption duration, days, median (range)	3 (1 – 9)	2 (1 – 7)	3 (1 – 9)	3 (1 – 8)	0.82
Hospitalization during CCRT, n (%)					
No	721 (90.7)	229 (92.0)	260 (90.6)	232 (89.6)	0.46
Yes	74 (9.3)	20 (8.0)	27 (9.4)	27 (10.4)	
Acute grade 3–4 toxicity, n (%)					
No	536 (67.4)	171 (68.7)	194 (67.6)	171 (66.0)	0.71
Yes	259 (32.6)	78 (31.3)	93 (32.4)	88 (34.0)	

Abbreviations: CARWL, C-reactive-protein-to-albumin ratio and weight loss; RT, radiotherapy; CCRT, concurrent chemoradiotherapy.

*RT interruption duration is reported only for the 89 patients who experienced RT interruption.

Table S2. Sensitivity multivariable Cox regression analysis for overall survival.

**Variable**	**Category / comparison**	**HR**	**95% CI**	**P-value**
Age group	>70 vs. ≤70 years	1.03	0.86 – 1.22	0.83
Sex	Male vs. female	1.06	0.79 – 1.38	0.72
ECOG performance status	1 vs. 0	1.09	0.82– 1.44	0.79
Smoking history	Present vs. absent	1.14	0.93 – 1.37	0.32
Histology	SCC vs. AC	1.04	0.94 – 1.17	0.68
T stage	T4 vs. T3	1.23	1.08 – 1.42	0.009
Chemotherapy cycles	Overall comparison	0.84	0.63 – 1.12	0.24
2 cycles	vs. 1 cycle	0.91	0.76 – 1.24	0.32
3 cycles	vs. 1 cycle	0.81	0.58 – 1.04	0.08
Radiotherapy interruption	No vs. yes	0.94	0.82 – 1.07	0.19
CARWL score group	Overall comparison	1.38	1.18 – 1.63	< 0.001
CARWL-1	vs. CARWL-0	1.34	1.16 –1.55	< 0.001
CARWL-2	CARWL-0	1.42	1.21 – 1.68	< 0.001
NPS group	Overall comparison	1.28	1.09 – 1.45	0.006
NPS-1	vs. NPS-0	1.17	1.03 – 1.34	0.008
NPS-2	vs. NPS-0	1.33	1.16 – 1.49	< 0.001

Abbreviations: AC, adenocarcinoma; CARWL, C-reactive-protein-to-albumin ratio and weight loss; CI, confidence interval; ECOG, Eastern Cooperative Oncology Group; HR, hazard ratio; NPS, Naples Prognostic Score; SCC, squamous-cell carcinoma.
